# Supplementary material for: Lattice thermal transport in two-dimensional alloys and fractal heterostructures
Source: Sci Rep. 2021 Jan 18;11:1656. doi: 10.1038/s41598-021-81055-4 (PMC7813883; doi:10.1038/s41598-021-81055-4)
Supplement: Supplementary file 1 — Supplementary Information. [file 41598_2021_81055_MOESM1_ESM.pdf]

Supporting Information for Lattice Thermal Transport in Two-Dimensional Alloys and Fractal Heterostructures

Aravind Krishnamoorthy[1], Nitish Baradwaj[1], Aiichiro Nakano[1], Rajiv K. Kalia[1], and Priya Vashishta[1,*]

[1]Collaboratory for Advanced Computing and Simulations, University of Southern California, Los Angeles, CA 90089
[*]priyav@usc.edu

1 Simulation Details

1.1 Molecular Dynamics Simulations

In these simulations periodic boundary conditions are applied to all directions with a vacuum of about 1000 Å in the z direction. The thickness of the monolayers are fixed at about half their vertical lattice constant which is about 6.47 Å[1] for $MoSe_2$ and WSe_2. NEMD simulations are performed with the newly parameterized SWFF using the LAMMPS molecular dynamics program[2]. The simulations are performed on rectangular monolayer single-crystals of dimensions L × L with periodic boundary conditions along the x and y directions. Prior to the imposition of a steady state for thermal conductivity measurements, the monolayer system undergoes an energy minimization step, where ionic positions are relaxed using the Stillinger Weber forcefield until there is no residual stress on the system. The system is then heated under the NPT ensemble to the desired target temperature where thermal conductivity is to be measured. Next, as defined in Figure 1 in the manuscript, two thin (≈100 Å wide) regions denoted as Hot and Cold, which span the entire width, L, of the simulation cell are defined at x=L/2 and x=3L/2. To establish a thermal steady state, a fixed energy flux, Q, is added to the kinetic energy of atoms in the Hot and region and an identical energy flux is removed the kinetic energy of atoms in the Cold region. This method[34] allows us to impose arbitrarily small thermal fluxes, in contrast to the momentum-swapping method of reverse non-equilibrium molecular dynamics[5], which can lead to large temperature gradients spanning 100 K and non-linear temperature profiles near the heat source and sink.

At steady state, this technique establishes a gradient in local temperature between L/2 and 3L/2 with an approximate and small temperature gradient of 10^{-3} K/Å , which falls within the linear regime for Fourier law of heat conduction[6]. This simulation scheme naturally captures both the mass variations and force-constant variations in alloyed and heterostructure systems that are responsible for phonon broadening etc[7]

The MD simulations use a time-step of 2 femtoseconds. Conjugate Gradient relaxation is done initially to obtain the correct box size. Following which the atoms are given a Gaussian distribution of velocities equivalent to 300K and the system is thermalized in a NVE ensemble for 20 picoseconds. This process is repeated 5 times. The system is then thermalized at 300K in a NVT ensemble for 200 picoseconds after which the system is ready for carrying out thermal conductivity calculations.

$$\Phi_H = -\kappa \frac{\partial T}{\partial x} \tag{1}$$

where Φ_H is the heat flux in the x direction and $\frac{\partial T}{\partial x}$ is the temperature gradient in the x direction. κ is the Thermal Conductivity.

The heat flux is given by,

$$\Phi_H = \frac{E}{A.\Delta t} \tag{2}$$

The temperature profile of the system is recorded every 1 nanosecond for 24 nanoseconds of the total simulation. This is done by dividing the system uniformly in the x direction into 320 bins of 20 Å each. The kinetic energy of the bin is used to calculate the temperatures using the energy Equipartition equation.

$$\langle E \rangle = \sum_{1}^{N} \frac{1}{2} m_i v_i^2 = \frac{3}{2} N k_b T \tag{3}$$

where v_i is the velocity of the i th atom and N is the total number of atoms in the bin.

1.2 Lattice Dynamics Calculations

Lattice dynamics simulations were performed in GULP[8] sing the same SW forcefield as MD simulations.

1.3 Stillinger Weber Forcefield

Accurate calculation of thermal conductivity requires an accurate potential to describe the forces between atoms. Here a Stillinger Weber(SW) potential has been employed because of its ability to describe both the stretching and bending of bonds. As in pervious studies[9–12] our forcefield reproduces the inter-atomic interactions in the covalently bonded 2H crystal through the 2-body and the 3-body energy terms. These model both bond bending and bond stretching. The total energy of the system in the SW framework is given by

$$E(r_1,r_2,...,r_N) = \sum_{i<j} V_2(r_{ij}) + \sum_{i<j<k} V_3(r_{ij}, r_{ik}, \theta_{i,j,k}) \tag{4}$$

The V_2 and V_3 terms represent the two and three body terms which have the following mathematical form.

$$V_2 = A\left(\frac{B}{r^p - 1}\right)e^{\frac{\gamma}{r-r_c}} \tag{5}$$

$$V_3 = Ke^{\frac{\gamma_1}{r_{ij}-r_0}+\frac{\gamma_2}{r_{jk}-r_0}}(\cos\theta - \cos\theta_0)^2 \tag{6}$$

where λ, γ_1, γ_2 are optimizable parameters. It is also interesting to note the harmonic dependence of energy on the bond angle θ. The two and three body terms decay exponentially for large values of r_{ij}. The short range nature of the forcefield makes it very easy to implement it numerically. To accurately model the anharmonic phonon phonon interaction[13], the forcefield includes energies that are dependent on atomic displacements up to the third order.

The SW forcefield is optimized using the multi-objective forcefield parameterization software, EZFF. Specifically, two-body parameters for Se-Se, Mo-Se, Mo-Mo, W-Se, W-W, Mo-W and three-body parameters for Se-Mo-Se, Mo-Se-Mo, Se-W-Se, W-Se-W, Mo-Se-W terms are optimized to reproduce multiple DFT-derived quantities relevant to thermal conduction in the Mo-W-Se system. The in-plane lattice constant, in-plane elastic modulus, C_{11}, and phonon dispersion along the Γ-M and Γ-K directions at three lattice strains $\varepsilon = -0.03, 0.0, 0.03$ are computed for four systems, undoped $MoSe_2$, undoped WSe_2 and $Mo_{0.25}W_{0.75}Se_2$ alloy and $(Mo|W)Se_2$ heterostructure. We use the non-dominated sort genetic algorithm, 3rd generation[14] to optimize the forcefield against these 6 objectives (phonon dispersions of 4 systems, average of lattice constants of the 4 systems, average of moduli of the 4 systems) for 2000 epochs. The optimized forcefield reproduced phonon dispersion curves to within 0.15 THz of the DFT-computed values and the lattice constants and elastic moduli are accurate to within 0.2 Å and 4.6 GPa respectively.

2 Computed lattice strain in alloys, fractals and superlattices

Table 1. Alloys and superlattice heterostructures have minimal lattice strain compared to $MoSe_2$ and WSe_2

System	Area per formula unit (Å)	Lattice Strain (%)
Pure MoSe2	9.377	0.0
3.7% Alloy	9.375	-0.015
7.41% alloy	9.374	-0.021
20.99% alloy	9.369	-0.0470
29.79% alloy	9.366	-0.0626
37.61% alloy	9.364	-0.072
Fractal Level 4	9.374	-0.019
Superlattice 3 divisions	9.375	-0.016
Superlattice 4 divisions	9.375	-0.016

References

1. Böker, T. *et al.* Band structure of mos 2, mose 2, and α- mote 2: Angle-resolved photoelectron spectroscopy and ab initio calculations. *Phys. Rev. B* **64**, 235305 (2001).
2. Plimpton, S. Fast parallel algorithms for short-range molecular dynamics. *J. computational physics* **117**, 1–19 (1995).
3. Ikeshoji, T. & Hafskjold, B. Non-equilibrium molecular dynamics calculation of heat conduction in liquid and through liquid-gas interface. *Mol. Phys.* **81**, 251–261 (1994).
4. Wirnsberger, P., Frenkel, D. & Dellago, C. An enhanced version of the heat exchange algorithm with excellent energy conservation properties. *The J. chemical physics* **143**, 124104 (2015).
5. Müller-Plathe, F. Reversing the perturbation in nonequilibrium molecular dynamics: An easy way to calculate the shear viscosity of fluids. *Phys. Rev. E* **59**, 4894 (1999).
6. Liu, X., Zhang, G., Pei, Q.-X. & Zhang, Y.-W. Phonon thermal conductivity of monolayer mos2 sheet and nanoribbons. *Appl. Phys. Lett.* **103**, 133113, DOI: 10.1063/1.4823509 (2013). https://doi.org/10.1063/1.4823509.
7. Körmann, F., Ikeda, Y., Grabowski, B. & Sluiter, M. H. Phonon broadening in high entropy alloys. *npj Comput. Mater.* **3**, 36 (2017).
8. Gale, J. D. Gulp: A computer program for the symmetry-adapted simulation of solids. *J. Chem. Soc. Transactions* **93**, 629–637, DOI: DOI10.1039/a606455h (1997).
9. Hong, Y., Zhang, J. & Zeng, X. C. Thermal conductivity of monolayer mose2 and mos2. *The J. Phys. Chem. C* **120**, 26067–26075 (2016).
10. Jiang, J.-W., Park, H. S. & Rabczuk, T. Molecular dynamics simulations of single-layer molybdenum disulphide (mos2): Stillinger-weber parametrization, mechanical properties, and thermal conductivity. *J. Appl. Phys.* **114**, 064307 (2013).
11. Norouzzadeh, P. & Singh, D. J. Thermal conductivity of single-layer wse2 by a stillinger–weber potential. *Nanotechnology* **28**, 075708 (2017).
12. Varshney, V. *et al.* Md simulations of molybdenum disulphide (mos2): Force-field parameterization and thermal transport behavior. *Comput. Mater. Sci.* **48**, 101–108 (2010).
13. Cahill, D. G. *et al.* Nanoscale thermal transport. ii. 2003–2012. *Appl. Phys. Rev.* **1**, 011305 (2014).
14. Deb, K. & Jain, H. An evolutionary many-objective optimization algorithm using reference-point-based nondominated sorting approach, part i: Solving problems with box constraints. *IEEE Transactions on Evol. Comput.* **18**, 577–601 (2014).
